# Supplementary material for: The Effects of Mitochondrial Transplantation on Sepsis Depend on the Type of Cell from Which They Are Isolated
Source: Int J Mol Sci. 2023 Jun 14;24(12):10113. doi: 10.3390/ijms241210113 (PMC10299019; doi:10.3390/ijms241210113)
Supplement: Supplementary file 1 [file ijms-24-10113-s001.zip › ijms-2407232-supplementary.pdf]

Supplementary Materials

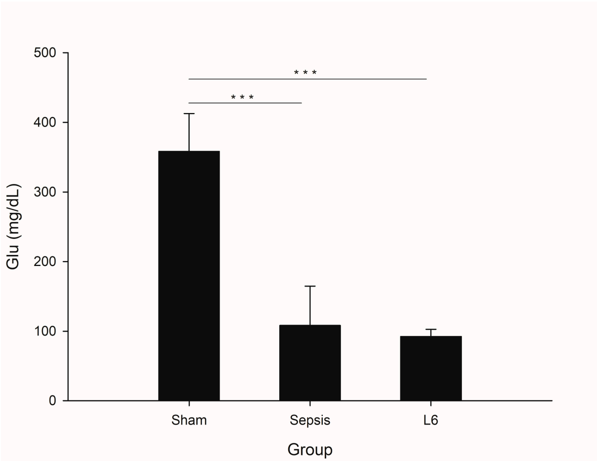

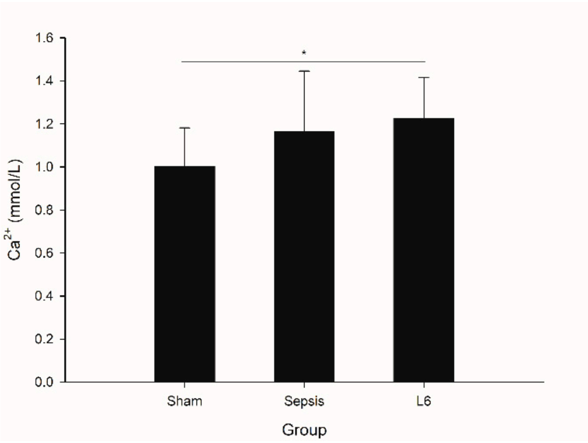

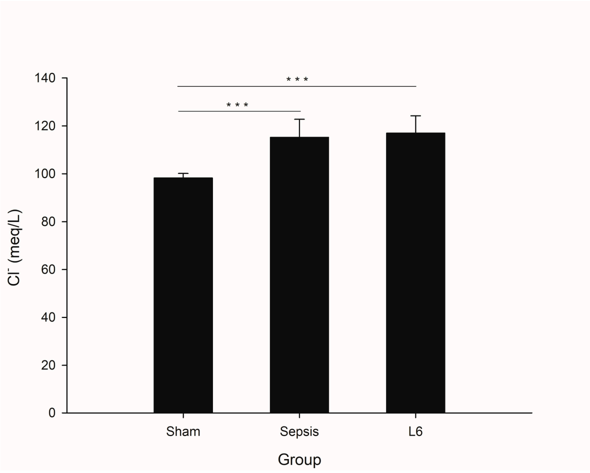

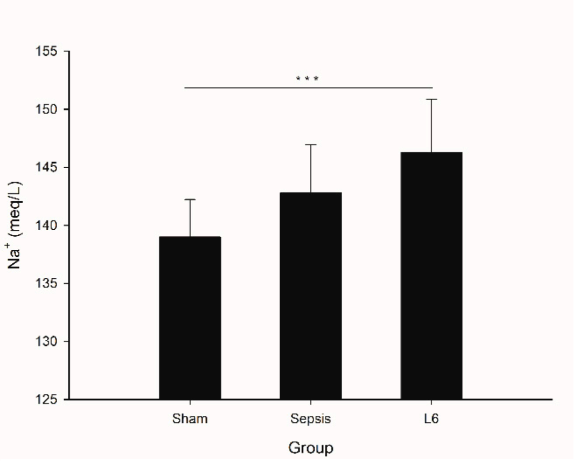

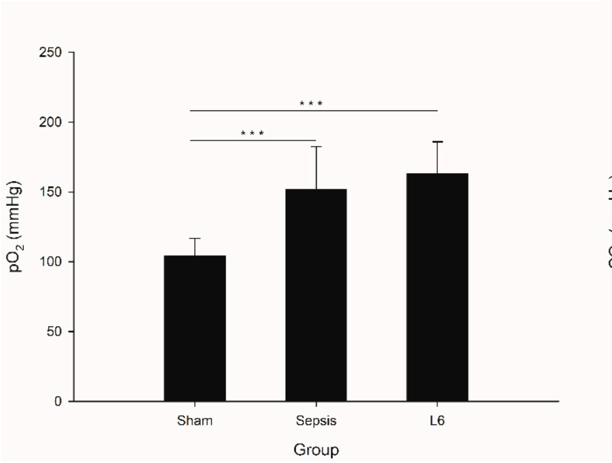

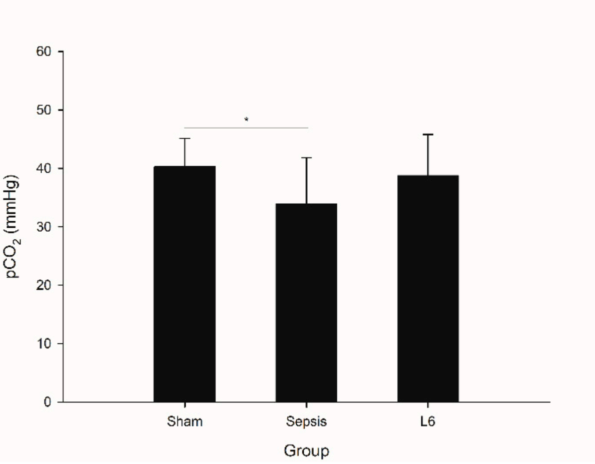

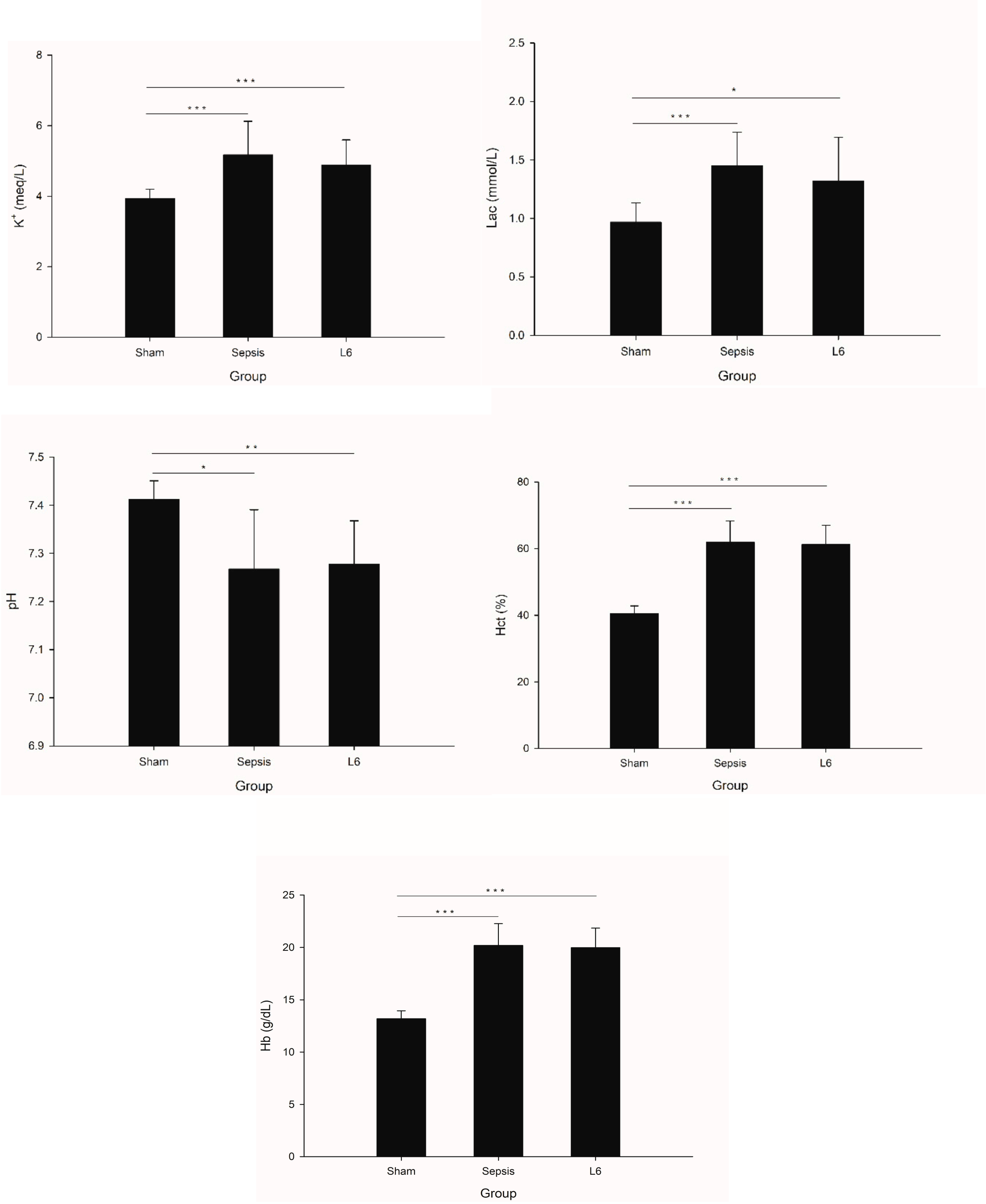

Supplementary Figure S1. Arterial blood gas analysis of in vivo fecal slurry model with mitochondrial transplantation (L6). Blood samples were analyzed at 24h after fecal slurry administration. The isolated mitochondria from L6 were injected into septic a rat after 1 h of sepsis induction. The results shown were the mean ± SD. Statistical analysis was performed using One-way ANOVA and * p < 0.05, ** p < 0.01, *** p < 0.001.
